# Racial Disparities in the Diagnosis and Prognosis of ALS Patients in the United States

**DOI:** 10.1007/s40615-024-02099-6

**Published:** 2024-07-25

**Authors:** Jaime Raymond, Theresa Nair, Kelly Graham Gwathmey, Theodore Larson, D. Kevin Horton, Paul Mehta

**Affiliations:** 1 Office of Analytics and Innovation, Agency for Toxic Substances and Disease Registry/Centers for Disease Control and Prevention, 4770 Buford Hwy, Atlanta, GA 30341 USA; 2 Department of Neurology, Virginia Commonwealth University, Richmond, VA USA

**Keywords:** Amyotrophic lateral sclerosis, Race, Minority populations, Clinical characteristics of ALS patients

## Abstract

**Background:**

Amyotrophic lateral sclerosis (ALS) is a progressive, fatal disease with largely unknown etiology. This study compares racial differences in clinical characteristics of ALS patients enrolled in the National ALS Registry (Registry).

**Methods:**

Data from ALS patients who completed the Registry’s online clinical survey during 2013–2022 were analyzed to determine characteristics such as site of onset, associated symptoms, time of symptom onset to diagnosis, and pharmacological and non-pharmacological interventions for White, Black, and other race patients.

**Results:**

Surveys were completed by 4242 participants. Findings revealed that Black ALS patients were more likely to be diagnosed at a younger age, to have arm or hand initial site of onset, and to experience pneumonia than were White ALS patients. ALS patients of other races were more likely than White ALS patients to be diagnosed at a younger age and to experience twitching. The mean interval between the first sign of weakness and an ALS diagnosis for Black patients was almost 24 months, statistically greater than that of White (*p* = 0.0374; 16 months) and other race patients (*p* = 0.0518; 15.8 months). The mean interval between problems with speech until diagnosis was shorter for White patients (6.3 months) than for Black patients (17.7 months) and other race patients (14.8 months).

**Conclusions and Relevance:**

Registry data shows racial disparities still exist in the diagnosis and clinical characteristics of ALS patients. Increased recruitment of non-White ALS patients and better characterization of symptom onset between races might aid clinicians in diagnosing ALS sooner, leading to earlier therapeutic interventions.

## Introduction

Amyotrophic lateral sclerosis (ALS) is a progressively fatal disease of which the actual pathogenesis and cause(s) remain largely unknown [1]. Recently studies have shown ALS in the United States (U.S.) to be more commonly diagnosed in White males over 60 years of age [2]. Previous epidemiologic studies addressing racial variation in ALS diagnosis and clinical characteristics have been limited [3, 4]. Global ALS prevalence rates vary widely ranging from 4.1 per 100,000 persons in Norway [5] to 8.4 per 100,000 persons in North-Eastern Italy [6, 7]. The most recent prevalence report in the U.S. estimated an adjusted prevalence rate of 9.1 per 100,000 persons [2]. One population-based study in the southeast part of the U.S. found a prevalence rate of 3.04 per 100,000 persons for the Black population [8].

Clinical characteristics vary widely among patients (e.g., site of onset, progression of the disease) [9]. The diagnosis of ALS can be challenging, with the site of onset resulting in several different phenotypes that include limb-onset, bulbar/respiratory-onset, or trunk/global onset [10]. Persons with bulbar onset typically have shorter life expectancy versus those with limb onset [11]. Patients typically experience various symptoms during the course of the disease such as muscle cramps, twitching (fasciculations), problems with speech (dysarthria), and difficulty swallowing (dysphagia) [12]. To date, only 10% of ALS cases are familial; the remaining cases are considered sporadic [13]. Studies have suggested that genetic, environment, and lifestyle factors play a significant role in ALS occurrence and phenotype [14–17].

The purpose of this paper is to compare racial differences in clinical characteristics of ALS patients enrolled in the U.S. National ALS Registry (Registry). Because ALS onset and progression among minority races have not been studied widely, these data provide additional information on phenotypic differences in a national population [12, 17, 18]. Having a better understanding of ALS among non-White patients regarding onset and progression might aid clinicians in making a quicker diagnosis, which could lead to earlier therapeutic interventions and help narrow the disparities, especially access to care, in those populations.

## Methods

### The National ALS Registry

In October 2010, the Agency for Toxic Substances and Disease Registry (ATSDR), part of the Centers for Disease Control and Prevention (CDC), launched the congressionally mandated, population-based National ALS Registry to help clarify the epidemiology of ALS in the U.S. [19]. The Registry’s purpose is to quantify the incidence and prevalence of ALS in the U.S., describe the patient demographics, and examine potential risk factors [20]. Details about the Registry’s objectives are presented elsewhere [21] as are its methods [22]. From there, the national administrative databases and the web portal are merged and de-duplicated to ensure that individuals are not counted twice. To verify ALS status within the web portal, ATSDR adopted the six questions from the U.S. Department of Veterans Affairs ALS registry that have been proven to be reliable indicators for accurate ALS diagnoses [23]. The Registry’s web portal also allows participants to complete brief online surveys about their ALS experience on topics such as occupational and military histories, smoking and alcohol use, and clinical symptoms. Currently, the Registry has 18 survey modules.

### Demographic Survey Module

The demographic survey module was one of the first six modules created for ALS patients to take part in. This module launched on October 19, 2010, when the Registry was initiated. Race was defined by standard federal definitions. For this analysis, the categories are White, Black, and other race. If more than one race was chosen, participants were categorized as other race. Body mass index (BMI) was calculated using a standard formula: BMI = weight (lb) / [height (in)]^2^ × 703 [24]. Other selected demographic characteristics for those who completed the clinical survey module were abstracted, including sex, ethnicity, age at diagnosis, and year registered.

### Clinical Symptoms Survey Module

The clinical symptoms survey module was created in partnership with the ALS Research Group, which includes U.S. and Canadian neurologists and researchers. The purpose of the module is to examine physical symptoms that participants developed before and after an ALS diagnosis. The survey contains 54 questions and covers topics such as the site of onset, time of initial symptom onset to diagnosis, and time from diagnosis to hospice referral. This module was launched in November 2013 for new Registry enrollees. Previous enrollees were prompted to return to the web portal to complete this survey.

### Data Analysis Methods

Site of onset refers to the body part to which a participant reported their first ALS-related weakness or symptom. The body was divided into 2 groups: (1) limb, which included extremities (hand, arm, foot, or leg), and (2) bulbar, which included speech or swallowing, or trunk/global, which included neck, back or abdominal areas, breathing muscles, or total body weakness. “Other symptoms” were those participants experienced after the initial symptom and could have occurred before or after diagnosis. The occurrence of symptoms was determined by calculating the time from symptom onset to diagnosis. Bivariate analyses were performed to examine the associations of race, symptoms following initial onset, and interventions for ALS. Categorical variables were assessed with chi-square tests. Statistical significance was considered at alpha = 0.05. All data analyses were performed using SAS 9.4 [25].

## Results

From the launch of the Registry in October 2010 through December 31, 2022, 10,894 adults aged 18 years or older self-enrolled via the Registry’s online portal and completed at least one of the 18 surveys. Of these persons, 4242 (38.9%) completed the clinical survey module, which didn’t launch until November 1, 2013, as well as the basic demographic survey module. Of those who responded to both surveys, 96% self-reported as White. Only 73 patients self-reported as Black, and 92 patients were classified as other races (e.g., Chinese, Native American). In the other race category, the majority self-reported themselves as Asian (62%). Table 1 lists the demographic characteristics of these 4242 patients, stratified by race. More than 26% of the Black patients were diagnosed with ALS before age 50 years, compared with only 12.9% of White patients *(p* = 0.0067). That was also true for a higher percentage of patients of other races (19.6% vs 12.9%, *p* = 0.0070). Other race patients also had a higher percentage of being at a normal BMI at the time of registration than did White ALS patients (44.6% vs. 34.7%, *p* = 0.0453). Lastly, other race ALS patients were less likely to have served in the military, compared with White ALS patients (8.7% vs. 18.3%, *p* = 0.0176). Black race patients did not see the same differences as other race patients.

**Table 1:** Demographic characteristics among U.S. adults with ALS who responded to the National ALS Registry’s clinical survey module, by race (November 1, 2013–December 31, 2022)

Characteristic	White patients		Black patients		Chi-square*	Other race patients		Chi-square^
	***N* = 4077**	**%**	***N* = 73**	**%**	***p*-value**	***N* = 92**	**%**	***p*-value**
**Age at diagnosis (years)**					0.0067			0.0070
18–49	525	12.9	19	26.7		18	19.6	
50–59	1,125	27.6	21	28.0		35	38.0	
60–69	1,582	38.8	23	32.0		29	31.5	
70 +	845	20.7	10	13.0		10	10.9	
**Sex**					0.2682			0.8048
Male	2,385	58.5	38	52.0		55	59.8	
Female	1,692	41.5	35	48.0		37	40.2	
**BMI at age 40 years**					0.0904			0.2972
Underweight/normal	1,321	33.7	16	23.9		34	39.1	
Overweight/obesity	2,595	66.3	51	76.1		53	60.9	
**BMI at registration**					0.9710			0.0453
Underweight/normal	1,400	34.5	25	34.7		41	44.6	
Overweight/obesity	2,656	65.5	47	65.5		51	55.4	
**Military history**					0.1483			0.0176
No	3,323	81.7	54	75.0		84	91.3	
Yes	746	18.3	18	25.0		8	8.7	
**Year registered**					0.3603			0.7213
Before 2014	510	12.5	10	14.0		9	9.8	
2014–2016	1,596	39.2	33	45.0		35	38.0	
2017–2019	1,042	25.6	12	16.0		23	25.0	
2020–2022	929	22.8	18	27.0		25	27.2	

^*^White patients are the referent group being compared to Black patients

^White patients are the referent group being compared to other race patients

Table 2 shows the site of onset among the 4242 patients, stratified by race. All patients who answered the question regarding the site of onset reported having progressive muscle weakness before the ALS diagnosis. More than 70% of the patients (*n* = 3015) had limb onset. A significantly higher proportion of Black patients had hand or arm onset weakness compared to White patients (*p* = 0.0012). Almost 29% of the 4242 patients experienced bulbar or global onset; of those, Black patients were slightly less likely to have bulbar or global onset than were White patients (*p* = 0.0690).

**Table 2:** Initial site of onset among U.S. adults with ALS who responded to the National ALS Registry’s clinical survey module, by race (November 1, 2013–December 31, 2022)

Initial site of onset^a^	White patients		Black patients*		Chi-square *p*-value	Other race patients^		Chi-square *p*-value
	***n***	**%**	***n***	**%**		***n***	**%**	
Limb	2,893	71.0	58	79.5	0.1126	64	69.6	0.7709
Arm or hand	1,383	33.9	38	52.1	0.0012	31	33.7	0.9638
Leg or foot	1,510	37.0	20	27.4	0.0906	33	35.9	0.8186
Bulbar or global	1,178	28.9	14	19.2	0.0690	28	30.4	0.7472

^a^Initial site of onset refers to the first body region where a patient reported a weakness or symptom prior to ALS diagnosis

^*^White patients are the referent group being compared to Black patients

^White patients are the referent group being compared to other race patients

Table 3 shows some of the other symptoms experienced by ALS patients. The most frequent symptoms during the course of the disease included muscle cramps (57.5%), twitching (56.4%), and problems with speech (35.6%). Among the 4242 patients, 24.9% had difficulty swallowing, and 22.6% experienced trips or falls. Almost 13% of participants had difficulty controlling bowels, 12.2% had experienced pneumonia, and 5.6% experienced blood clots. Compared with White patients, when stratified by race, a higher proportion of Black patients reported suffering from pneumonia (*p* = 0.0067) and those of other races saw a slightly higher proportion of twitching (*p* = 0.0582).

**Table 3:** Other symptoms experienced^a^ among U.S.adults with ALS who responded to the National ALS Registry’s clinical survey module, by race (November 1, 2013–December 31, 2022)

Other symptoms	White patients		Black patients		Chi-square *p*-value*	Other race patients		Chi-square *p*-value^	Total	
**(Have you ever experienced the following?)**	***n***	**%**	***n***	**%**		***N***	**%**		***n***	**%**
Pneumonia	218	5.4	10	13.9	0.0067	4	4.4	0.8490	232	5.6
Falls	917	22.6	18	24.7	0.8619	25	27.5	0.5077	960	23.2
Cramps	2,271	55.9	45	61.6	0.5838	59	64.1	0.2405	2,375	57.5
Twitching (fasciculations)	2,224	54.8	47	64.4	0.2433	60	65.9	0.0582	2,331	56.4
Problems with speech (dysarthria)	1,419	35.0	19	26.0	0.2639	32	35.2	0.4048	1,470	35.6
Difficulty swallowing	1,019	25.1	13	17.8	0.3588	25	27.5	0.1952	1,057	25.6
Difficulty controlling bowels	479	11.8	14	19.2	0.1126	12	13.2	0.1282	505	12.2

^a^The following symptoms could have occurred before or after an ALS diagnosis and regardless of site of onset

^*^White patients are the referent group being compared to Black patients

^White patients are the referent group being compared to other race patients

**Fig. 1:**
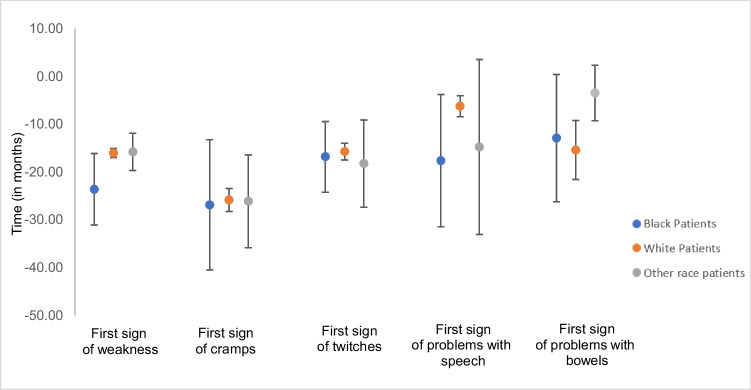
Mean time from ALS diagnosis to symptoms onset stratified by race among U.S. adults with ALS, November 1, 2013–December 31, 2022

The time between symptom onset and ALS diagnosis was calculated for each symptom and stratified by race (Fig. 1). White and other race patients saw muscle weakness onset approximately 16 months before an ALS diagnosis; Black patients showed a mean time of almost 24 months before diagnosis (*p* = 0.0374). Of the 1470 patients with onset of speech problems, the mean time to ALS diagnosis was approximately 6 months for White patients, about 16 months for Black patients, and 18 months for other race patients. Bowel issues preceded an ALS diagnosis by approximately 3 months for patients of other races, but almost 13 months for White patients, and 15 months for Black patients.

**Fig. 2:**
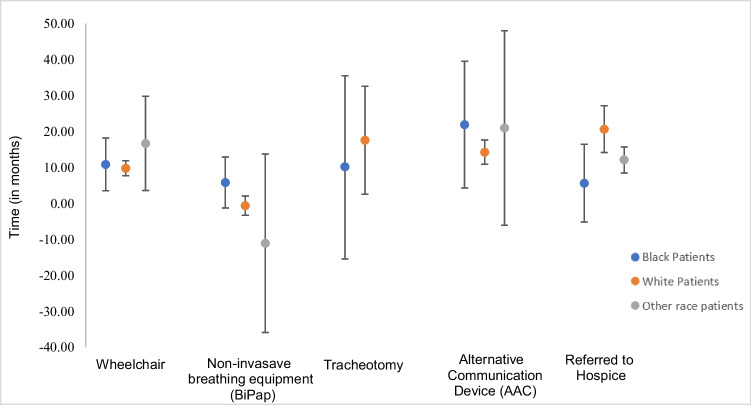
Mean time from ALS diagnosis to specific interventions stratified by race among U.S. Adults with ALS, November 1, 2013–December 31, 2022

Figure 2 shows the use of non-pharmacological interventions, stratified by patient race. Other race patients commonly used noninvasive breathing equipment much sooner, with a mean time of 11 months before an ALS diagnosis, compared to White patients at 1 month before diagnosis and Black patients at 6 months after their ALS diagnosis. For tracheostomies, White patients had a mean time of almost 18 months after diagnosis, whereas Black patients had a mean time of 10 months after their ALS diagnosis. We did not have stable data for patients of other races. On average, Black ALS patients were referred to hospice earlier than the other groups. Black patients had a mean time from diagnosis to hospice of less than 6 months. The mean time from diagnosis for patients of other races was 12 months, and for White patients, it was almost 21 months.

## Discussion

Health disparities for minority populations are an ongoing societal problem that is also reflected in differential access to care. Our findings represent a large national cohort. As in other regional or clinic-specific studies, our findings show that Black patients and those of other races have a different ALS disease course than do White patients [26–28]. Although White patients as a group were diagnosed earlier, the diagnostic delay for all ALS patients remains long, with a reported average of approximately 12 months [29, 30]. Our data show this delay is more evident in the minority cohort within the Registry, especially for Black patients. White patients with a bulbar onset were likely to be diagnosed the earliest. Conversely, Black patients were more likely to be diagnosed at a younger age (< 50 years) than were patients of any other racial group [27]. Age of onset and progression is likely influenced by genetic polymorphisms, ethnicity, and environmental exposures [31–33].

It is not yet known why Black ALS patients tend to have earlier disease onset, though identification of genetic modifiers is an active area of research. Our finding that problems with the bowels occurred 15 months before diagnosis in Black ALS patients may help providers detect ALS earlier. Similarly, it is not known why noninvasive ventilation was found to begin an average of 11 months before diagnosis for other race patients, but it could indicate that difficulty breathing may be one of the first symptoms for this population. Communicating these findings to providers may assist with earlier diagnosis across races.

Our analyses also showed a phenotypic variation: upper limb onset was more common among Black patients than among other racial groups. The reason is unknown, but the small number of Black patients may bias the sample. In the future, it may be possible for the Registry to form stronger partnerships with groups serving Black communities to increase representation through registration.

BMI was another factor of interest in our cohort. Other studies have shown that a higher BMI is associated with a longer survival time [34]. In general, Black persons have a higher prevalence of obesity than do White persons or other racial groups, and we saw similar results in BMI at age 40 for White patients vs. Black patients [35]. Although not calculated in our analyses, Black patients generally have a longer survival than White patients [28]. This might partially be attributed to higher rates of tracheostomy and invasive ventilation among Black patients [28]. Race might not be an independent predictor of survival time if controlled for age of onset, bulbar onset, and *C9orf72* gene positivity, the most frequent genetic mutation leading to ALS [27]. This higher level of BMI, overweight or obese, could be a protective factor in ALS as shown by our analyses and other studies [36].

This study is subject to several limitations. First, the data are not a random sample from the database, and it is likely that some biases are introduced by the self-identification process. There are many reasons why patients might not enroll in the registry and complete surveys, such as lack of access to the necessary technology and internet to do so, lack of awareness of the registry, and concerns related to data security and exposure of personal health identification. Another limitation is the small sample size for Black and other race ALS patients. This makes it difficult to generalize the findings of the analyses. One of the most striking findings in this study is the low number of Black registrants who took the symptoms survey. Of the 4242 registrants who took the symptom survey, only 73 (1.7%) were Black. From recent epidemiological studies, approximately 6.5% of ALS patients in the U.S. are predicted to be Black [2, 4]. Clearly, we are missing a considerable amount of Black ALS patients in the Registry; therefore, selection bias might affect these findings. There are only 264 Black ALS patients who completed the demographics survey through 2022. In reviewing all Black ALS patients who completed the demographics survey, we did see similar results in age and sex compared to those who completed the demographics as well as the symptoms onset survey. Black patients were significantly more likely to be diagnosed at a younger age compared to White ALS patients (*p* < 0.0001) but no significance in sex. The ALS community and advocates must encourage participation in the National ALS Registry across all racial and ethnic groups to ensure the most comprehensive demographic and natural history sample possible, as well as access to clinical trials.

These data show how ALS clinical characteristics differ widely by race, but a larger representation of Black patients and other race patients is needed for further research. An ALS diagnosis is primarily based on clinical assessment; therefore, a diagnosis is often deferred to specialists with expertise in this rare condition after several months of testing. Treatment of ALS, as for other diseases, is not immune from racial disparities. More initiatives are needed to raise awareness about the signs and symptoms of ALS to improve access to care for minority populations. Educating physicians about the disparities in access to care for minority ALS populations and the timing of non-pharmacological interventions might help lengthen survival for all ALS patients.

## Data Availability

The data are available via CDC—Amyotrophic Lateral Sclerosis: Research Application Form.
